# Uncovering the genetic mechanism of rind color trait in watermelon using fine mapping and comparative transcriptomic analysis

**DOI:** 10.3389/fpls.2025.1553166

**Published:** 2025-03-13

**Authors:** Sitong Liu, Sikandar Amanullah, Bohan An, Yu Guo, Xiaoxue Liang, Xiujie Liu, Jixiu Liu, Yue Gao, Wen Zhao, Chengzhi Yuan, Meiling Gao

**Affiliations:** ^1^ College of Life Science, Agriculture and Forestry, Qiqihar University, Qiqihar, China; ^2^ Department of Horticultural Science, North Carolina State University, Mountain Horticultural Crops Research and Extension Center, Mills River, NC, United States; ^3^ Heilongjiang Provincial Key Laboratory of Resistance Gene Engineering and Protection of Biodiversity in Cold Areas, Qiqihar, China; ^4^ Qiqihar Agricultural Technology Extension Center, Qiqihar, China

**Keywords:** 2-phyto-1, 4-β-naphthoquinone methyltransferase protein, fine mapping, rind color, transcriptome, watermelon

## Abstract

The rind color of watermelon fruit is a significant trait that directly affects consumer acceptability. However, the genetic regulatory mechanisms underlying rind color remain poorly understood. In this study, we crossed two differentiated watermelon lines (K2Q “female parent line with a light green rind” and K2S “male parent line with a dark green rind”) and developed segregated F_2_ mapping populations. The dynamic development of rind color was observed by identifying the critical period for color transformation as occurring between 7 and 14 days after pollination (DAP). Genetic segregation analysis indicated that a single dominant gene regulates the major genetic locus (*ClRC*) associated with the dark green rind trait. Whole-genome BSA-sequencing (BSA-seq) and fine mapping analysis exposed the delimited *ClRC* locus to a 37.52 kb region on chromosome 08 (Chr08), comprising five genes. The pairwise sequence comparisons analysis of the parental lines revealed the single major gene (*Cla97C08G161570*), which encodes a 2-phytyl-1,4-*β*-naphthoquinone methyltransferase protein, exhibiting one non-synonymous type single nucleotide polymorphism (nsSNP) at candidate site (Chr8:27994761, C-G). The real-time quantitative polymerase chain reaction (RT-qPCR) verified the higher expression level of the K2S line on the 14 DAP than that of the K2Q line. The analysis of comparative transcriptomes (RNA-sequencing) identified a total of 940 differentially expressed genes (DEGs) associated with rind coloration in the two parental lines at three dynamic stages of development (0, 7, and 14 DAP). Gene Ontology (GO) and Kyoto Encyclopedia of Genes and Genomes (KEGG) enrichment analysis revealed key genes (*C01G023430*, *C04G071470*, *C09G165830*, *C07G128820*, *C08G148460*, and *C08G155040*) that share the same pathway as the *Cla97C08G161570* gene and exhibited high levels of differential expression trend. Further, RT-qPCR verified that these genes display the same expression pattern as the *Cla97C08G161570* gene, and expression levels in the dark green rind lines were significantly higher than those in the light green rind lines, suggesting the significant role in modulating the pigmentation activity.

## Introduction

Watermelon (*Citrullus lanatus* L.) is a significant fruit crop of the Cucurbitaceae family. It is divided into different sub-species of the main species, e.g., (1) *C. lanatus* (Thunb.) Matsum. & Nakai, (2) *C. amarus* Schrad., and (3) *C. mucosospermus* Fursa (Fursa, 1972; Renner et al., 2014; Paris, 2015). China is the largest producer of watermelon and ranks first among the top ten producers in the world (Amanullah et al., 2023). It has become an excellent model plant for dissecting the essential biological pathways involved in the regulation of numerous complex traits. To date, extensive breeding studies have been performed to understand the genetic basis of divergent traits of watermelon for the development of improved cultivars (Pan et al., 2020).

The rind color is an important aesthetic trait that influences consumer choices, and most of the edible fruits exhibit a wide genetic diversity of rind and flesh colors (Kim et al., 2010; Borovsky et al., 2013; Amanullah et al., 2023). The color variation in plant organs is attributed to the composition and relative abundance of different types of photosynthetic pigments; however, the main photosynthetic pigments that influence the rind color of horticultural crops include chlorophyll, phycobilin, carotenoids, anthocyanins, and flavonoids (Matsumoto et al., 2004). There is a positive correlation between rind color variation and the biological pathway of chlorophyll biosynthesis; however, the significant expression of several associated genes (*CHLH*, *HEMA1*, and *GUN4*) is known to be light-dependent and regulated by circadian rhythms (Peter and Grimm, 2009). It was identified that *ClaAPRR2* is associated with the dark green rind color of watermelon (Oren et al., 2019). In light green watermelon, a mutation in the intron leads to the premature termination of the translation of its transcript. Further, research has indicated that the metabolism of secondary metabolites can influence leaf color shade transformation (Xu et al., 2020). The candidate DEGs have significant contributions in four major pathways: metabolic pathways, biosynthesis of secondary metabolites, ribosome function, and photosynthesis, and genes help in the modulation of the pigmentation process (Liu et al., 2023).

Chlorophyll (Chl) is the key biological factor influencing the green color of Cucurbitaceae and other crops (Lancaster et al., 1997). However, many studies have examined color changes in fruits, leaves, and other plant tissues, providing valuable insights into chlorophyll metabolism and chloroplast formation (Liu et al., 2007). In rice, the potential gene (*CSP41b*) has been identified for maintaining normal leaf pigmentation and structural morphology of chloroplasts (Mei et al., 2017). In tomatoes, the *SlSGR* gene mainly regulates the chlorophyll degradation process in fruits. In its natural mutant form (gf), *SlSGR* is either artificially silenced or knocked out in wild-type fruits. The expression of *SlSGR1* significantly inhibits the chlorophyll degradation process, resulting in mature fruits with an outer appearance of a rusty red color due to the accumulation of undegraded chlorophyll (Uluisik et al., 2022). Further, differences in chloroplast morphology and chlorophyll content have been observed in tomato fruit with dark and light green rinds. The *SlMYB72* gene was found to inhibit the expression of *SlTKN2*, *SlCHLH*, and *SlPOR1*, thereby affecting chloroplast development and synthesis, and silencing of *SlMYB72* led to a significant increase in chlorophyll content in tomato fruits (Wu et al., 2020). Additionally, the *WGL2* gene, encoding a conserved ribosomal protein, was found to regulate formation of chloroplasts in rice, and mutations in this gene caused disruption in expression across several metabolic processes, including photosynthesis process, biosynthesis of chlorophyll, formation of chloroplasts and ribosomes, ultimately resulting in an albino phenotype (Qiu et al., 2018).

Watermelon rind color is categorized into nine distinct types: yellow, dark yellow, light yellow, green-white, light green, yellow-green, green, dark green, and pure dark green. Since the 1930s, a genetic model for watermelon rind traits has been gradually established. The segregating populations derived from different parental sources were used to demonstrate the candidate single dominant gene (*G*) modulating the dark green rind color (Weetman, 1937). It has been reported that the three alleles “*G* (dark-green rind), *gs* (light-green stripes), and *g* (light-green rind)” are mainly responsible for regulating the watermelon rind surface color and stripe color patterns; however, the *gs* allele is recessive to the *G* allele yet dominant over *g* (Poole, 1944). The advanced genetic inheritance model of watermelon rind and stripe color was proposed, e.g., *G*, *gW* (wide stripes on the rind), *gM* (medium-wide stripes on the rind), *gN* (narrow stripes on the rind), and *g*, with the dominant effect ranked as *G* > *gW* > *gM* > *gN* > *g* (Lou and Wehner, 2016). The solid green rind was identified as completely dominant over the striped light green (*gs*) and partially dominant over the unique type of light green, gray or yellowish green (Yang et al., 2015).

It was stated that dark green color is dominant over light green color of rind (Kumar and Wehner, 2011). The *Dgo* gene controls yellow peel, while the *g* gene controls light green peel, which is dominant over the dark green gene (*G*). The green stripes (*gs*) are recessive, and *g-1* and *g-2* can also influence light green peel (Xiao, 2013); however, the *gs*-regulated green striped rind is recessive to dark green but dominant over light green, and the *D* gene controlling dark green rind and the *Dgo* gene regulating yellow rind of watermelon was found to be located at the front genetic segment of chromosome 4 (Park et al., 2016), and fine mapping delimited this region to a 59.80 kb interval (Dou et al., 2018). A precise location of the key gene regulating rind color was also identified within a 31.40 kb interval on chromosome 8 by using high-density genetic mapping, and the *ClCG08G017810* was predicted as a candidate gene (Li et al., 2019). The additional potential genes (*Cla97C09G175170* (a two-component response regulator-like protein, *APRR2*) and *Cla97C03G066110*) were pinpointed by using a segregated mapping population (Luan et al., 2022). Recently, the primary genetic mapping also revealed that light green rind is governed by main-effect genetic locus, positioned within a 1.25 Mb interval on chromosome 9 (Amanullah et al., 2023).

In addition, the publicly available reference genome assemblies significantly assisted genetics and breeding studies of watermelon (Guo et al., 2013; Wu et al., 2019). The bulked segregant analysis-based sequencing (BSA-seq) approach helped in rapid detection and fine mapping of the main-effect genetic locus harboring the candidate functional genes governing several differentiated phenotypes (Li et al., 2017; Yang et al., 2022; Mi et al., 2023). Transcriptome sequencing (RNA sequencing, RNA-seq) can simultaneously detect the expression levels of a large number of genes. This approach has been widely applied for the identification of differentially expressed genes (DEGs) involved at the transcriptional and translational levels of the regulatory pathways of many significant traits of melon and watermelon (Zhu et al., 2017; Liu et al., 2023; Yang et al., 2024).

The genetic regulatory mechanisms controlling rind color of many Cucurbitaceae plants have been reported very well, but the studies on identifying the complex genetic mechanism controlling rind color of watermelon materials with different genetic backgrounds did not get good attention. In the existing study, we conducted whole genome sequencing, bulked segregant sequencing analysis, and comparative transcriptomic analysis (RNA-sequencing) to perform fine genetic mapping as well as identify key genes regulating differentiated rind color traits in the developed segregation mapping population derived from contrasted parent lines.

## Materials and methods

### Experiment materials and mapping population

**Figure 1:**
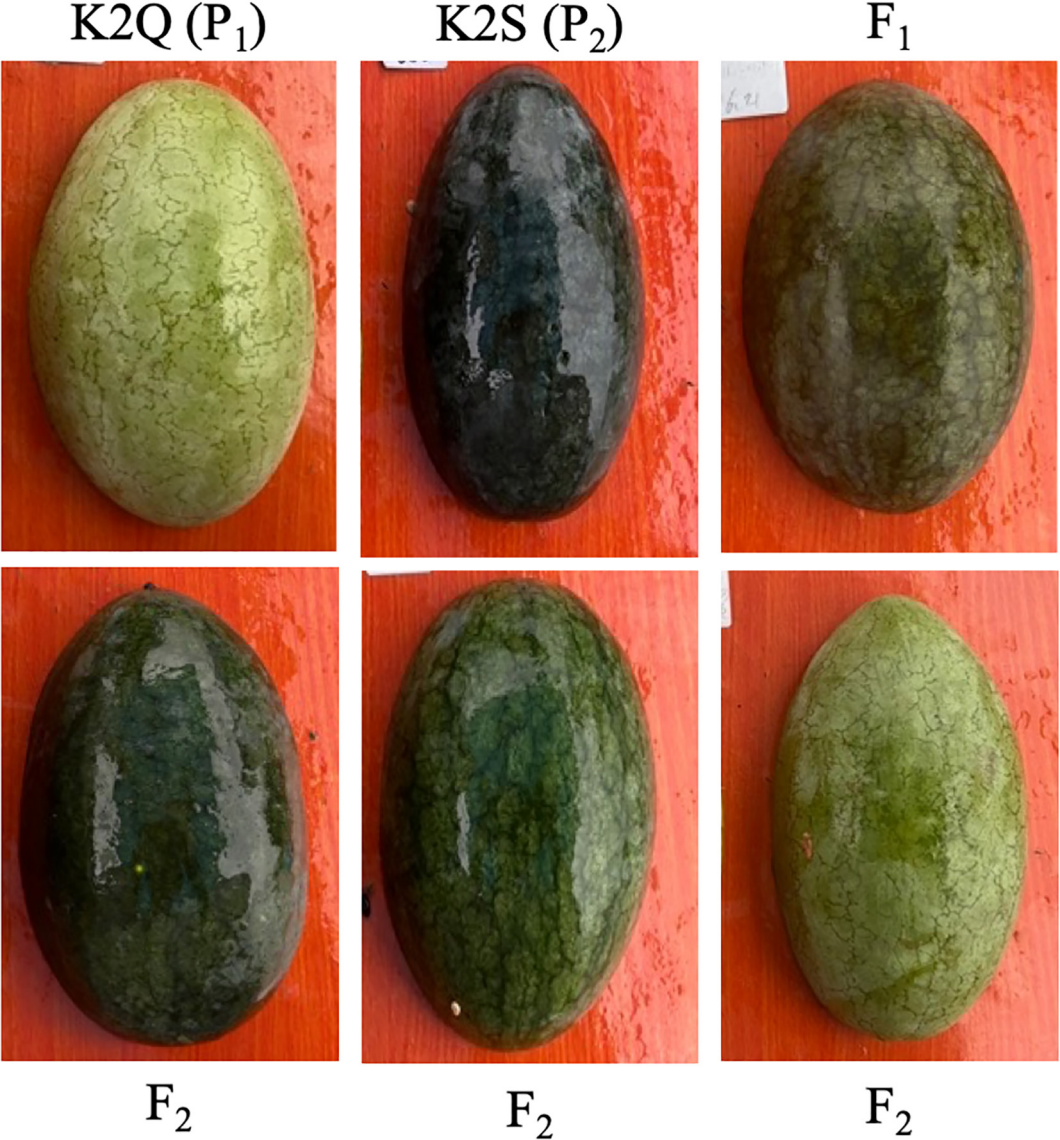
The visual rind phenotypes exhibiting differentiated color pattern among parental lines (K2Q and K2S), F_1_ (offspring), and F_2_ mapping population.

Two comparative watermelon lines “K2Q (P_1_, the female parent line with light green rind color on fruit surface and K2S (P_2_, the male parent line with dark green rind color on fruit surface)” and natural panel of GWAS accession were selected as experimental materials (**Figure 1**, **Supplementary Table S1**), respectively. The basic visual differences in the fruit rind color on the parent lines and acquired mapping populations are shown in **Figure 1**. The plants of each population were cultivated at the Qiqihar Agricultural Technology Extension Center, Qiqihar, China. These both lines were crossed to obtain the segregated F_2_ mapping populations, respectively.

In 2022, a total of 12 plants of each P_1_, P_2_, and F_1_ hybrid were grown using three repetitions of each, and a primarily segregated F_2_ population (n=121) was successfully cultivated and used for the preliminary detection of the candidate genetic loci (*ClRC*) modulating watermelon rind color. In 2023, an extended F_2_ mapping population (n=861) was developed and utilized for fine genetic mapping of detected *ClRC* loci and contributing genes, respectively. Furthermore, a plant population comprising a natural panel of GWAS accessions of watermelon was cultivated for relative assessment of the experiment. All mapping populations were cultivated on raised ridges within a controlled environment of a greenhouse using a randomized complete block design (RCBD) for planting geometry. The plant-row spacing of 45×55 cm on bed ridges was used, vines were hung, single vines were pruned, second female flower-fruit retention was practiced, and all typical procedures were used to raise healthy crop plants.

### Phenotyping of rind color

The dynamic changes in the rind surface color were observed to identify the critical transition period from light to dark green color, based on the first day after pollination (DAP) to 30 days of fruit development and maturity. This observation also served as the basis for determining the optimal sampling period for the analysis of transcriptome sequencing and quantitative real-time polymerase chain reaction. The bloomed flowers were hand-pollinated, and one watermelon fruit was harvested at proper maturity after observing the characteristics of the rind surface color in the parental lines and mapping population. Fruit rind color was classified into dark and light green appearances based on visual scoring (0 and 1), respectively. The recorded phenotypic dataset was computed in Microsoft Excel (version 2019), and genetic inheritance was checked by the chi-square analysis using IBM-SPSS software (version 26.0).

### Determination of photosynthetic pigment and histology of rind samples

The rind samples (0.2 grams for each sample) from fruits of both parent lines were collected at various developmental stages (7, 14, 21, and 28 DAP), and photosynthetic pigments (chlorophyll and carotene) were checked according to the earlier reported ethanol extraction method (Liu et al., 2023). The contents of chlorophyll a (Chla), chlorophyll b (Chlb), total chlorophyll (Chl), and carotene (Car) were estimated using three repetitions of each group, respectively.

The formulas used for these calculations were as follows:


Chla=(12.7A663nm−2.69A645nm)×V/(W×1000);

Chlb=(22.9A645nm−4.68A663nm)×V/(W×1000);

Car=(1000A470nm−3.27Chla−104Chlb)×V/(229×1000×W);

Chl=Chla+Chlb.

For the observation of developed chloroplast structure, the transmission electron microscope (TEM, Hitachi HT7700) was utilized to observe and photograph the chloroplasts after drying at room temperature, allowing for an examination of their structure and size (Liu et al., 2023).

### DNA extraction and whole genome BSA-seq

Total genomic DNA was isolated from young leaf tissues using an extraction buffer of cetyltrimethylammonium bromide (CTAB) (Allen et al., 2006). For the BSA-seq analysis, two DNA bulks of comparative fruit rind samples having light green and dark green colors were chosen from the primarily segregated F_2_ population, respectively. Whole-genome resequencing (20×) of two mixed pools and distinct parental lines was performed at the Beijing Genomics Institute (BGI) Company in Wuhan. The re-sequencing of 144 natural germplasm resources was also performed at BGI, and raw data is available in our laboratory (unpublished), which facilitated us for further bioinformatics analysis.

Then, all of the raw sequencing reads were then aligned, and high-quality reads were screened across the reference genome (V2_97103) of watermelon using the BWA software (Li and Durbin, 2009). The SNP variants were called by samtools and bcftools (Danecek et al., 2021), and the *delta* index of single nucleotide polymorphism was estimated according to the previously reported study (Li et al., 2017), where the delta SNP index of 1 and 0 indicates a high or no link between SNPs and the corresponding trait, individually. The locally estimated scatterplot smoothing (LOESS) regression analysis was used to assess for a significant connection (*P* ≤ 0.01). Finally, the area above the threshold line was identified as a candidate segment for the preliminary positioning of the rind color-regulating gene (Zhang et al., 2021). Further, the whole genome resequencing data of 144 natural germplasm resources of watermelon was also used for comparative validation of targeted loci and existing SNPs.

### Development of genetic markers

Firstly, the major genetic locus was primarily pinpointed using the BSA-seq method. Next, the InDels and dCAPS markers were generated in the identified major locus using the filtered clean end-sequenced reads of comparative parent lines. The primary InDel markers were developed based on the recently reported method (Mi et al., 2023), and dCAPS markers were identified using dCAPSFinder (version 2.0) based on the respective and suitable restriction enzymes (Kushanov et al., 2016), respectively. All relevant primer sequences were prepared using the default configuration of PCR primer design software “Primer Premier (version 6.25)” and oligo-synthesized. Then, all primers were marked with short abbreviations to facilitate the molecular genotyping experiments in segregated F_2_ populations. The information on forward and reverse primers, physical positions, product lengths, and endonucleases is mentioned in **Supplementary Tables S2**, **S3**, respectively.

### Fine mapping of targeted *ClRC* locus

For primary genetic mapping, the produced InDel markers were tested within the genotypes of the K2Q line, K2S line, and their F_1_, and a total of 19 codominant markers were used for genotyping using the F_2_ population (*n* = 121). Then, fine genetic mapping was carried out by delimiting the primary region of the *ClRC* locus based on dCAPS and InDels genotyping using an expanded F_2_ population (*n* = 864), which enabled us to determine the maximum marker coverage and expose the potential delimited region in kb intervals, as well as screen recombinant lines.

The amplification of polymerase chain reaction (PCR) for InDels and dCAPS markers was carried out as follows: e.g., 1.0 μl of each primer (forward and reverse), 1.0 μl of DNA, 94°C temperature for 5 min, 35 cycles of 94°C for 1 min, annealing temperature (Ta) for 45 sec, then 72°C temperature for 1 min. The yielded PCR products of genotyped markers were separated and visualized based on their size by running them on agarose gel, respectively. Then, the combined coded genotypic and phenotypic datasets were employed for evaluating the centimorgan (cM) positions of markers using the JoinMap software (version 4.0) (Ooijen, 2006).

### Prediction of candidate genes, expression, and phylogenesis

The candidate genes were predicted within the narrowed down interval on chromosome (**Supplementary Table S4**), and the functional annotation was checked by surfing the referenced genome of watermelon (V2_97103) and the online database of the National Center for Biotechnology Information (NCBI, https://www.ncbi.nlm.nih.gov/). The candidate genes that exist in the main chromosomal region were identified using the online Cucurbit Genomics Database (http://www.cucurbitgenomics.org/JBrowse/). The paired sequencing reads of comparative watermelon lines (K2S and K2Q) were checked for gene mutations using the versatile sequence analysis function of DNAMAN software (version 9.0). Then, the full-length gene sequence was obtained, and gene primers were exported and synthesized, respectively.

For the gene expression analysis, the required samples of fruit rind were chosen at dynamic stages of growth and development (0, 7, and 14 DAP) of differentiated genetic materials (K2S and K2Q). The high-quality RNA was isolated using the ready-to-use TRizol™ reagent technique (Rio et al., 2010), and first-strand complementary DNA was synthesized by following the user manual of the SureScript™ First-Strand cDNA Synthesis Kit. The specific primers (forward and reverse) of candidate genes were exported based on the open reading frame (ORF) sequence, an annealing temperature (Ta) of 60°C, and an amplification length between 200 to 300 bp. A SYBR Green-qPCR reaction mixture containing 0.3 µL of gene primer and 0.02 µL of cDNA was prepared, and the relative gene expression pattern was assessed using three biological and technical replications of each rind tissue sample based on Applied Biosystems StepOne™ and StepOnePlus systems and the delta-delta Ct (2^−△△CT^) method (Livak and Schmittgen, 2001). The *β-*Actin was used as a housekeeping gene to compare the expression of other target genes with a sample (Kong et al., 2014). The primer information of predicted candidate gene sequences (forward and reverse) used for RT-qPCR verification can be seen in **Supplementary Table S5**.

Further, the online database of UniProt (https://www.uniprot.org) was utilized to search for homologous amino acid sequences of potential genes found in 13 species, and the phylogenetic relationship of the ClRC protein was studied. ClustalW software was used to align protein sequences, and MEGA software (version 5.0, http://www.megasoftware.net/) was used to create a phylogenetic tree using bootstrap replications (1000×) and the maximum likelihood technique (Felsenstein, 1981). The suitable conserved domains were also pinpointed by scanning the online database of the NCBI website (https://www.ncbi.nlm.nih.gov/cdd).

### RNA sequencing, DEGs identification, and validation

For the RNA-sequencing (RNA-Seq), total RNA was isolated from rind samples collected at 0, 7, and 14 DAP from both parental lines using the RNfeasy Mini Kit (QIAGEN, Germany). RNA-seq libraries (300 bp) were developed and bioinformatic analysis were performed at Kidio Biotechnology Company, Guangzhou, as reported in our recent study (Yang et al., 2024). FastQC (version 1.0.0) was used to evaluate the quality of raw sequencing reads (Andrews, 2010); TopHat (version 2.1.1) was used to map all filtered clean-end reads (Trapnell et al., 2012); and HTSeq (version 2.0.3) was used to determine the final mapped reads of each transcript (Anders et al., 2015). The raw RNA-seq data reads (PRJNA1206455, named Watermelon Peel Background Color Transcriptome at Different Stages, with independent libraries and biological replicates) were uploaded to the Sequence Read Archive (SRA) database of the NCBI website (https://www.ncbi.nlm.nih.gov/search/sra/?term=PRJNA1206455).

The candidate DEGs across the randomized groups (G1, G2, and G3) were calculated using the edgeR software (version 4.0), as previously documented procedures (Yang et al., 2022; Lv et al., 2022; Yang et al., 2024). The candidate DEGs sets were screened by following the criteria of FoldChange ≥ 1.7 and FDR < 0.05. Then, the screened DEGs were utilized for GO and KEGG enrichment analysis. Further, we conducted transcription factor (TF) annotation on the target gene pool of DEGs associated with rind coloration by utilizing the transcription factor database (https://planttfdb.gao-lab.org). The primers of identified DEGs were designed, the relative expression levels of key genes were confirmed using RT-qPCR analysis, and significant differences were found using the techniques mentioned above. The statistical differences of expression trends between light green rind and dark green rind of fruits were verified through statistical analysis (Student’s t-test) using IBM-SPSS Statistics (version 29.0).

## Results

### Analysis of chloroplast development in fruit rind

**Figure 2:**
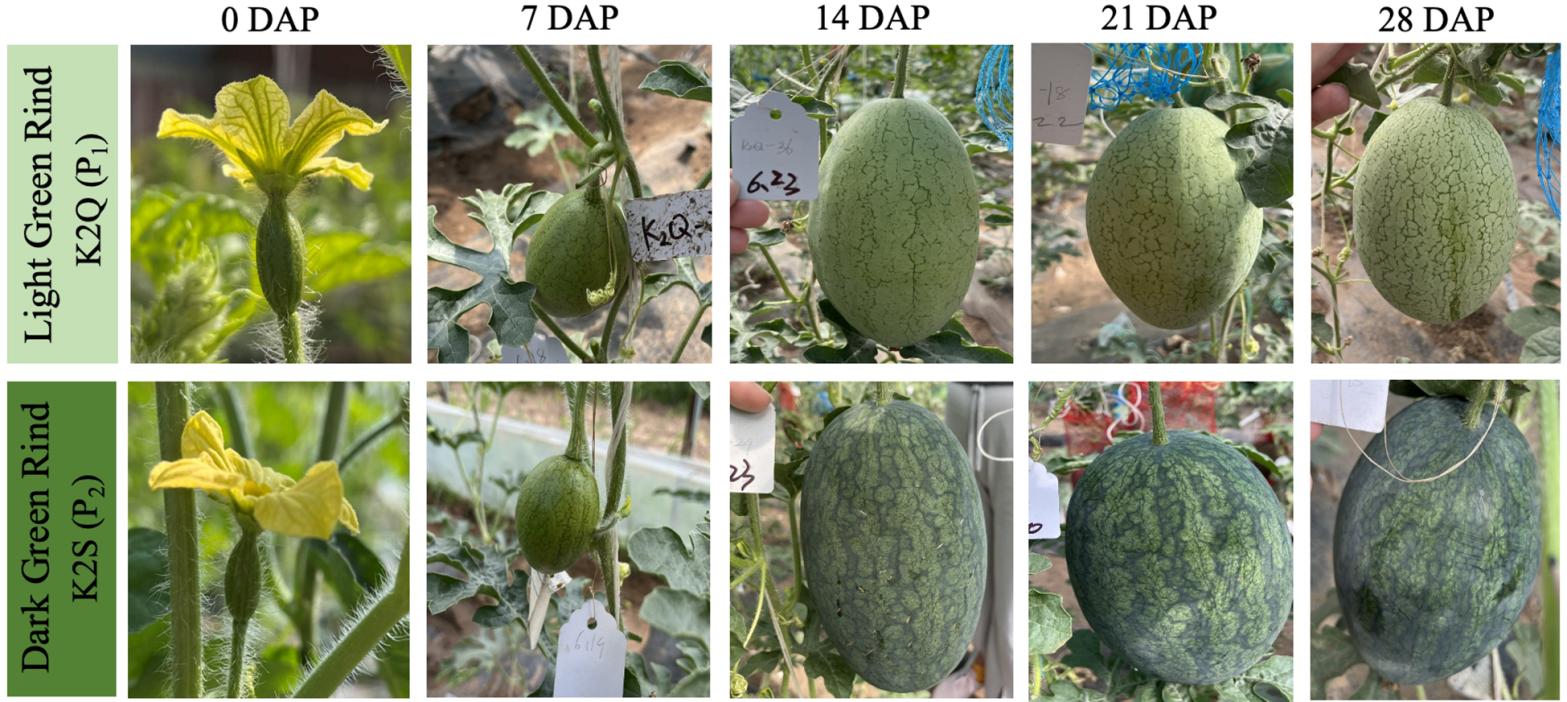
A pictorial view of dynamic changes in the rind color of watermelon grown at various developmental stages. DAP refers to days after pollination.

The dynamic changes in the rind color of contrasted parent lines (K2Q and K2S) were observed from the first day of flowering to 28 days after pollination (DAP), and visual and genetic differences in rind coloration were observed (**Figure 2**). There was no significant difference in the visual rind color, and no significant difference was observed in the photosynthetic pigments (mg/g) of the rind color of both parental lines at 0 and 7 DAP.

Further, it was noticed that the rind color of the K2S line gradually became darker at 14, 21, and 28 DAP, exhibiting higher total chlorophyll contents than that of the K2Q line, but there was no significant difference in carotene contents during the rind development at 7 DAP (**Table 1**). In contrast, the rind color of the K2Q line remained light green during all stages, and a less amount of chlorophyll and carotenoid contents was noticed than that of the K2S line (dark green rind). These observations indicated that the period from 7 to 14 DAP is critical, where biological and genetic regulatory mechanisms take place for triggering the change in rind surface color of watermelon fruit.

**Table 1:** Analysis of changes in photosynthetic pigment content in the fruit rind of contrasted parent lines grown at different developmental stages.

Photosynthetic pigments (mg/g)	Genetic materials	7 DAP	14 DAP	21 DAP	28 DAP
Chlorophyll	K2S (P_2_)	0.231 ± 0.05	0.910 ± 0.03**	0.841 ± 0.21**	0.957 ± 0.02**
	K2Q (P_1_)	0.243 ± 0.01	0.322 ± 0.06	0.186 ± 0.03	0.129 ± 0.14
Carotene	K2S (P_2_)	0.037 ± 0.03	0.151 ± 0.03	0.141 ± 0.02	0.170 ± 0.03
	K2Q (P_1_)	0.037 ± 0.04	0.056 ± 0.01	0.032 ± 0.06	0.023 ± 0.04

DAP refers to days after pollination. Asterisks symbols (**) are representing the significant results at *p* < 0.01.

**Figure 3:**
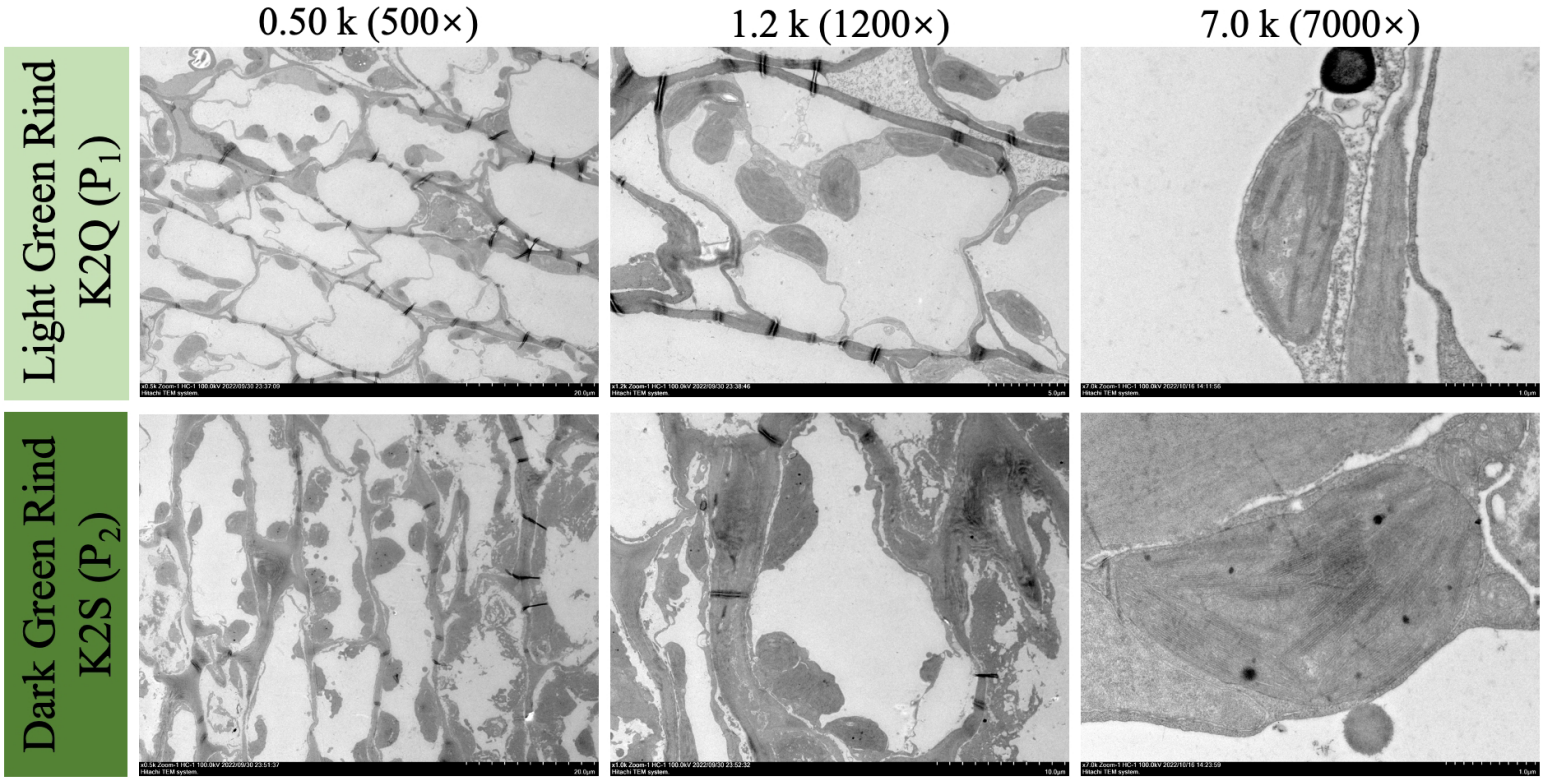
The visual observations of internal ultrastructure of developed chloroplasts in fruit rind of contrasted parent lines at 0 and 28 DAP, under different magnification scales of microscope (0.5k×, 1.2k×, and 7.0k×), respectively.

Further, the microscopic analysis revealed that the parent line (K2S) with dark green rind color exhibited more chloroplasts as compared to the parent line (K2Q) with light green rind color (**Figure 3**). These chloroplasts were not only larger in size but also exhibited the irregular shapes with a dense distribution that allows them to fit more closely together. Furthermore, the thickness of the thylakoid grana has increased significantly, demonstrating a more orderly arrangement and a higher degree of stacking.

### Analysis of genetic segregation of rind color

From the phenotypic data analysis, we noticed that the mature fruits of both parent lines (K2S and K2Q) showed comparable fruit rind color, and fruits of F_1_ progeny exhibited the dark green rind, consistent with the K2S line (**Figure 1**, **Table 2**). However, among the fruits of the segregated F_2_ population (n = 121), a total of 88 fruits showed dark green rind color, and 33 fruits showed light green color on the fruit surface, exhibiting a perfect Mendelian ratio (3:1, *χ^2^* = 0.33 and *P* > 0.05) (**Table 2**), and suggesting that dark green rind color in watermelon is controlled by a single dominant gene locus, named *ClRC*. Further, visual rind phenotypes and genomic sequencing data of the natural GWAS accessions of watermelon depicted candidate loci associated with variation in rind color (**Supplementary Table S1**).

**Table 2:** Analysis of genetic segregation of rind color in contrasted parents, F_1_ and developed segregated biparental F_2_ populations.

Populations	Total	Dark green rind	Light green rind	Segregation ratio	*χ^2^* value^a^	*P* value^b^
K2S (P_2_)	36	36	–			
K2Q (P_1_)	36	–	36			
F_1_	36	36	–			
F_2_	121	88	33	3:1	0.333	0.564

^a^χ^2^ > χ^2^, 0.05 = 3.841 and ^b^ *P* < 0.05 are indicating a significant difference.

### Analysis of primary mapping of *ClRC* locus

**Figure 4:**
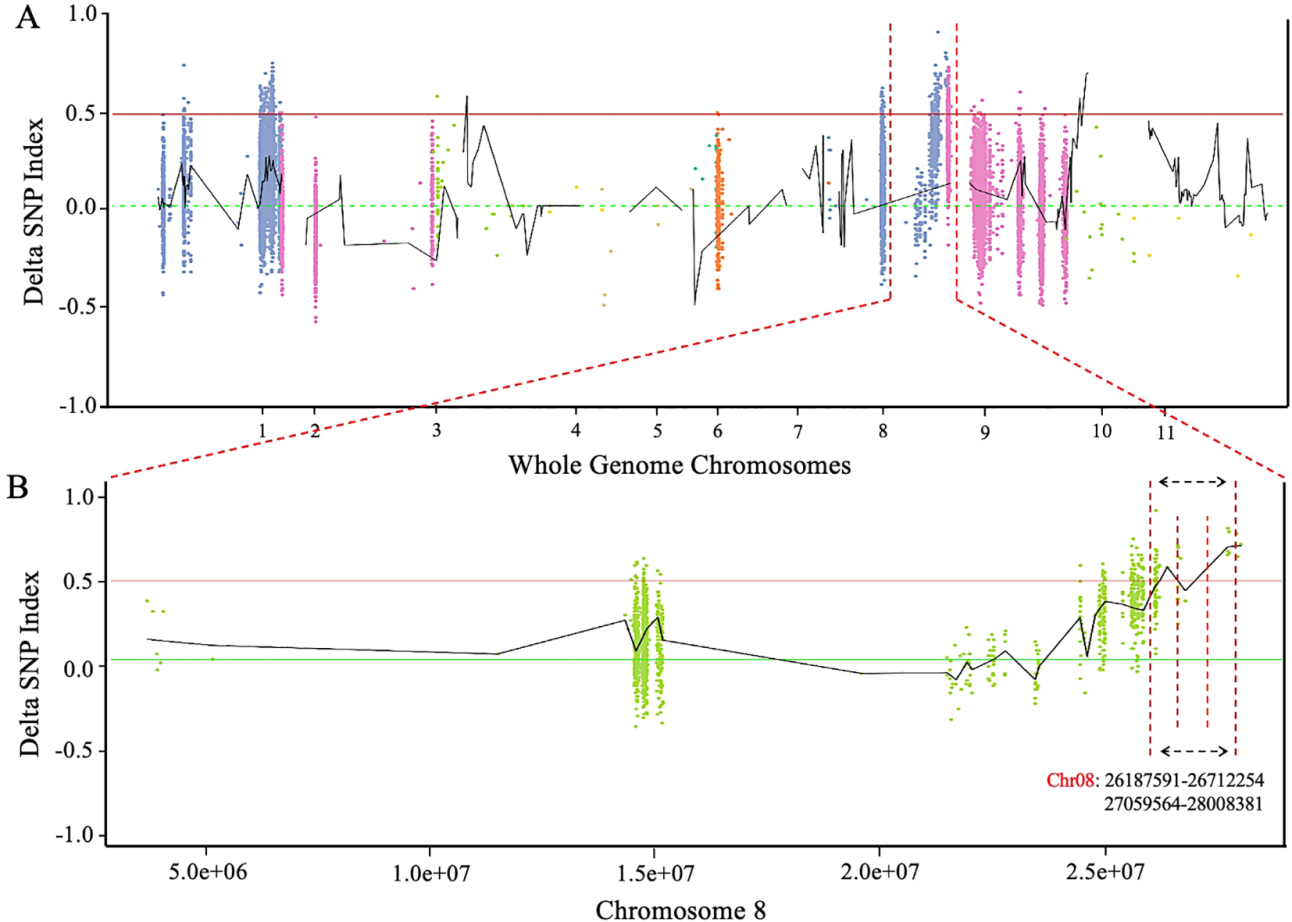
Detection of major genetic loci regulating rind color phenotype. **(A)** Delta SNP-index over watermelon chromosomes. **(B)** Delta SNP-index over chromosome 8 of watermelon genome. The maximum threshold is denoted by horizontal dotted interval lines (*P* < 0.01).

The resequencing data of two extreme pools with dark green and light green rinds were utilized to calculate the ΔSNP-index across the whole genome of watermelon. BSA-seq analysis identified a candidate segment that was located on chromosome 8 (**Figure 4A**), specifically at positions of 0.53 Mb and 0.94 Mb (Chr8: 26187195-26712254 and 27059564-28008381 bp) within two distinct blocks (**Figure 4B**). Furthermore, genomic sequencing data of a natural population of 144 germplasm accessions identified a clustered locus (*qfrc8.1*), spanning about 27,888,937 to 28,111,239 bp, or roughly 0.3 Mb. This region shows preliminary mapping overlap with the segments identified through BSA-seq.

### Analysis of fine mapping for candidate genes

**Figure 5:**
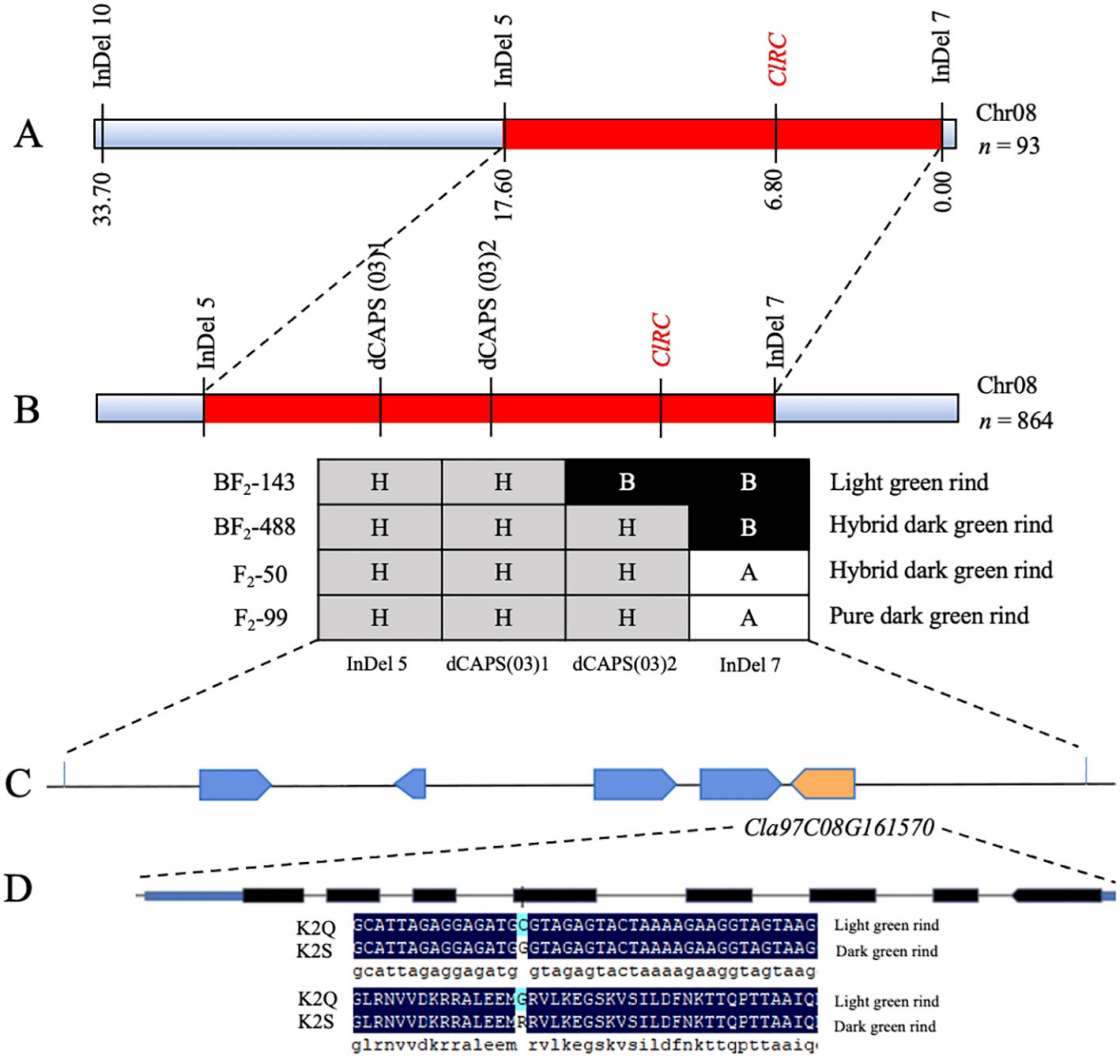
Fine mapping of the *ClRC* gene regulating rind color of watermelon. **(A)** Primary mapping of main-effect *ClRC* loci using the 93-F_2_ mapping population. **(B)** Delimiting of *ClRC* loci using an extended 864-F_2_ mapping population. **(C)** Identification of candidate *Cla97C08G161570* gene within the delimited segment. **(D)** Structure of *Cla97C08G161570* gene and non-synonymous mutations existence in the fifth exon.

Based on the candidate regions obtained from BSA-seq, molecular markers were developed at each ~50 kb interval within the targeted genetic interval. A total of ten InDel markers were developed for the main genetic regions of Chr8:26187195-26712254 and 27059564-28008381, respectively (**Supplementary Table S2**). Among the developed markers, InDel-5, InDel-7, and InDel-10 were identified as polymorphic between the parental lines and offspring through agarose gel electrophoresis. Genetic mapping was performed using an F_2_ population consisting of 93 individuals, and two flanking markers (InDel-5 and InDel-7) exhibited the *ClRC* gene locus at Chr8: 27948932 (**Figure 5A**). These candidate markers (InDel-7 and InDel-5) positioned at 0.00 cM and 17.60 cM were further utilized to test and genotype the expanded F_2_ generation population (n=864) planted in the next year of 2023 (**Figure 5B**). A total of 35 recombinant plants were screened, and candidates were further refined based on the physical distance between the flanking markers (InDel-5 and InDel-7), with an effort to identify SNP sites of every 5-10 kb for conversion into dCAPS markers. Ultimately, a total of 20 dCAPS markers were developed (**Supplementary Table S3**), with polymorphisms detected only between dCAPS(03)1 and dCAPS(03)2, which were employed for genotyping the selected recombinant individual strains. The *ClRC* gene was ultimately identified within a 37.52 kb region located between dCAPS(03)2 and InDel-7 (positions 27,968,227 to 28,005,749).

A total of five genes (*Cla97C08G161530*, *Cla97C08G161540*, *Cla97C08G161550*, *Cla97C08G161560*, and *Cla97C08G161570*) were annotated (**Figure 5C**) using the BLAST analysis. The *Cla97C08G161570* gene was predicted as a candidate gene because it similarly exhibited a non-synonymous SNP site (Chr8: 27,994,761, C (Cytosine) → G (Guanine)) in the fifth exon that also caused an amino acid mutation (R (arginine) to G (glycine)), respectively (**Figure 5D**). This gene is encoding the 2-phytyl-1,4-*β*-naphthoquinone methyltransferase, chloroplastic (**Supplementary Table S4**), and shows a critical part in the biosynthesis of chloroquinone and is assumed to be involved in the chlorophyll status and light reaction system. So, we hypothesized that the *Cla97C08G161570* gene is mainly involved in regulating the dark green rind in the K2S line of watermelon.

### Analysis of gene expression and phylogenetic association

**Figure 6:**
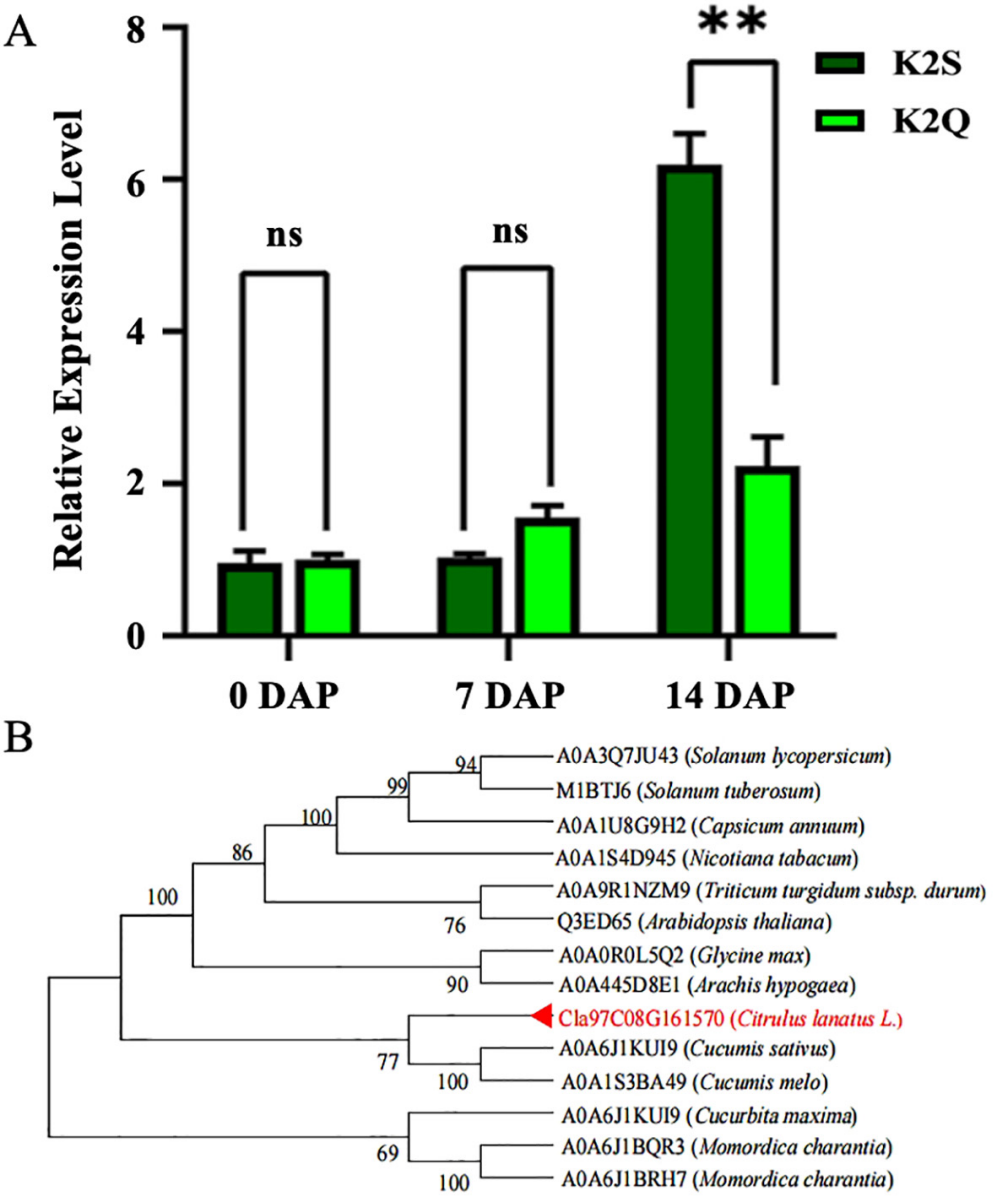
The expression level and phylogenesis of the *Cla97C08G161570* gene controlling differentiate rind color. **(A)** The gene expression level at various developmental stages of comparative parental lines. “ns” indicates non-significant difference and DAP refers to days after pollination. **(B)** Phylogenetic analysis of amino acid sequences of *MENG*-like genes. Note: The candidate gene is highlighted in red font, and the numbers adjacent to branch points indicate the reliability of those branches. Asterisks symbols (**) are representing the significant results at p < 0.01.

The quantitative reverse transcription PCR technology was employed to assess the expression levels of the *ClRC* gene (*Cla97C08G161570*) in the rind samples of the K2S and K2Q lines. The obtained results (**Figure 6A**) illustrated no significant differentiation in the expression levels of the *ClRC* gene between the K2S and K2Q lines at 0 and 7 DAP; however, the expression level in the K2S line was significantly higher at 14 DAP compared to the K2Q line, indicating that the *Cla97C08G161570* gene positively regulates the dark green rind color.

For understanding the phylogenetic relationship between the protein encoded by *Cla97C08G161570* and its homologs, we searched for homologous genes using the databases of NCBI and the UniProt website. It was noticed that the protein from the watermelon gene (*Cla97C08G161570*) shares a close connection with other Cucurbitaceae crops (**Figure 6B**), involving cucumber (*Cucumis sativus*), melon (*Cucumis melo*), winter squash (*Cucurbita moschata*), and turban squash (*Cucurbita maxima*). This gene also demonstrated conserved evolution within the Cucurbitaceae family. So, we compared the amino acid sequences of the homologous genes and identified a total of 13 genes that were highly homologous to the *ClRC* gene of watermelon (*Cla97C08G161570*), including those from tomato (*A0A3Q7JU43*), potato (*M1BTJ6*), pepper (*A0A1U8G9H2*), tobacco (*A0A1S4D945*), wheat (*A0A9R1NZM9*), Arabidopsis (*Q3ED65*), soybean (*A0A0R0L5Q2*), peanut (*A0A445D8E1*), cucumber (*A0A6J1KUI9*), melon (*A0A1S3BA49*), pumpkin (*A0A6J1KUI9*), and bitter melon (*A0A6J1BQR3* and *A0A6J1BRH7*). The obtained results exposed that the amino acid sequence lengths ranged from 233 to 277; however, the *Cla97C08G161570* gene exhibited a 67.90% sequence similarity with *MENG*-like gene in *Arabidopsis thaliana* and contained a methyltransferase chemotaxis domain. This domain demonstrates conservation, although amino acid mutations also occur within it. We utilized a total of 14 protein sequences and constructed a phylogenetic tree. The proteins from Cucurbitaceae crops clustered into one group, while those from Leguminosae and Solanaceae crops formed separate groups. The clustering of homologous sequences aligns with the known phylogenetic relationships among these species, with watermelon *Cla97C08G161570* being closest to cucumber and muskmelon.

### Analysis for DEGs screening and transcriptional factors

**Figure 7:**
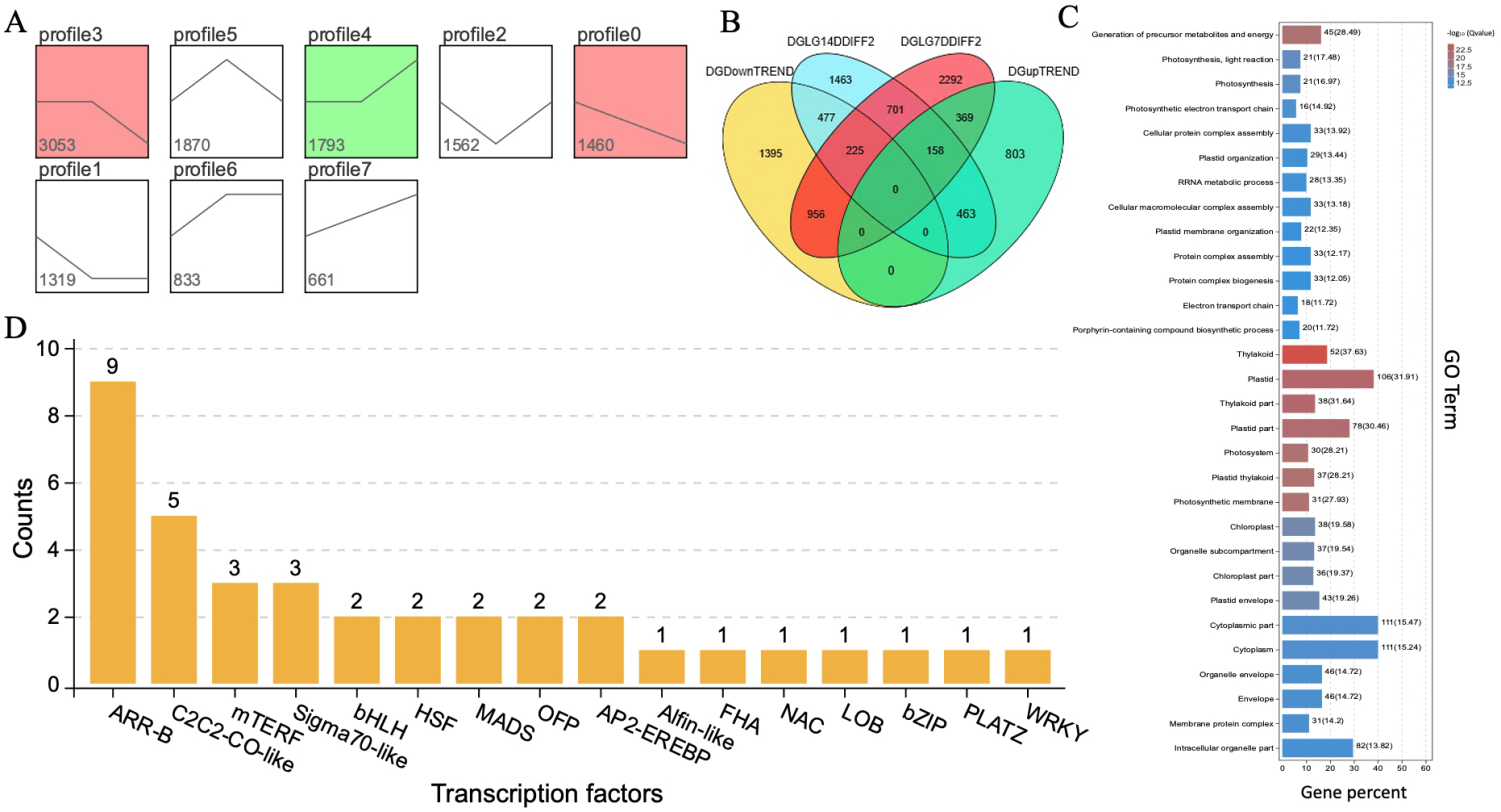
Identification of candidate DEGs and gene enrichment terms. **(A)** Clustering trend analysis. **(B)** VENN diagram of identified DEGs. **(C)** Identified GO terms. **(D)** Transcriptional factors, respectively.

For uncovering the regulatory mechanism of key genes and transcriptional factors contributing to the rind color formation, the transcriptome datasets analysis of rind samples of both parent lines (K2S and K2Q) was analyzed in three contrasted groups, including their subgroups (G1 (G1-1, G1-2, and G1-3), G2 (G2-1, G2-2, and G2-3), and G3 (G3-1, G3-2, and G3-3)) of different and same genetic materials within developmental stages, as shown in **Supplementary Table S6**, **Figure 7**, respectively.

In the G1 group of the same genetic material (K2S with a dark green rind), a comparison between the randomized stages of 0, 7, and 14 DAP showed that the G1-1 subgroup (DG_0D-vs-DG_7D) exhibited about 2800 DEGs that were identified from samples collected at 0 and 7 DAP, comprising 1987 up-regulated genes and 813 down-regulated genes. In the G1-2 subgroup (DG_0D-vs-DG_14D), a total of 6689 DEGs were detected in the samples of 0 and 14 DAP, comprising 2613 upregulated genes and 4076 downregulated genes, with 762 common differentially expressed genes identified. In the G1-3 subgroup (DG_7D-vs-DG_14D), a total of 6613 DEGs were pinpointed from samples collected on the 7th and 14th DAP, which included 2649 up-regulated genes and 3964 down-regulated genes. However, a total of 762 common genes were identified among all the subgroups (G1-1, G1-2, and G1-3) of the same genetic materials at the same development periods (**Supplementary Table S6**).

In the G2 group of two different genetic materials (K2S and K2Q), a comparison between the same developmental periods, the G1-1 subgroup (DG_0D-vs-LG_0D) depicted a total of 687 DEGs, comprising 388 genes with up-regulated expression and 299 genes with down-regulated expression. In the G1-2 subgroup (DG_7D-vs-LG_7D), about 4,701 DEGs were detected; among them, 1,826 genes were up-regulated and 2,875 genes were down-regulated. In the G1-3 subgroup (DG_14D-vs-LG_14D), a total of 3,487 DEGs were identified, including 2,011 genes with up-regulated expression and 1,476 genes with down-regulated expression. However, a total of 90 common genes were identified among all the subgroups (G2-1, G2-2, and G2-3) of different genetic material at the same development period (**Supplementary Table S6**).

In the G3 group of identical genetic material (K2Q line with a light green rind), a comparison between the randomized different developmental periods (0, 7, and 14 DAP) showed that the G3-1 subgroup (LG_0D-vs-LG_7D) exhibited a total of 6035 DEGs, comprising 2572 up-regulated genes and 3463 down-regulated genes. In the G3-2 subgroup (LG_0D-vs-LG_14D), a total of 6543 DEGs were detected, comprising 2527 upregulated genes and 4076 downregulated genes. In the subgroup of G3-3 (LG_7D-vs-LG_14D), a total of 4801 DEGs were pinpointed; among which, a total of 2117 DEGs showed upregulated expression and 2684 DEGs showed downregulated expression. However, about 1357 DEGs were identified as common among all the subgroups (G3-1, G3-2, and G3-3) of the same genetic materials at different development periods (**Supplementary Table S6**).

In addition, we performed gene expression analysis using a clustering method based on the characteristics of samples observed at three different stages of growth and development (0, 7, and 14 DAP) in the K2S line. The gene sets exhibiting specific biological characteristics were selected from the clustering results and trend analysis, as shown in **Figure 7A**. The field phenotypes analysis indicated that the period between 7 and 14 DAP is a critical transition phase for coloration. Similarly, clustering profile 3 exhibited no significant change in gene expression from 0 to 7 DAP but showed a significant downward trend from 7 to 14 DAP, while clustering profile 4, which demonstrated no significant change from 0 to 7 DAP but a significant upward trend from 7 to 14 DAP. So, we selected target genes to construct a gene pool comprising differentially expressed genes ((DEGs) regulating rind color. A critical period for color change was identified between 7 and 14 DAP.

The DEGs that intersect with the target gene set from profiles of the trend analysis were analyzed and identified by a Venn diagram (**Figure 7B**). We selected a target gene set that displayed no significant change in gene expression from 0 to 7 DAP, followed by an upward and downward trend in expression from 7 to 14 DAP. The Venn diagram of genes also allowed us to select the intersection of the DEGs set from the parental K2S and K2Q lines at 14 DAP, along with the ascending and descending trend gene sets, ensuring no overlap with the DEGs of the 7 DAP set from the same parental line. This resulted in two specific regions (477 and 463), which contained the differential genes from the target gene pool for DEGs related to rind coloration, including the candidate *ClRC* gene. The target gene pool of DEGs associated with rind coloration was classified according to Gene Ontology (GO) functions (**Figure 7C**). The obtained results showed that the categories of immune system processes, metabolic processes, and single-organism processes contained the largest number of DEGs; however, the candidate genes exhibited significant differences in their GO function entries, e.g., plastid, thylakoid, and production of precursor metabolites and energy (Generation of Precursor Metabolites and Energy). These particular molecular functions may play a role in regulating the rind coloring process.

Further, the transcription factor analysis determined whether each gene is a member of a specific transcription factor family and allowed us to summarize the number of genes within each TF family. As illustrated in **Figure 7D**, the transcription factor families with the highest number of annotated genes were *ARR-B*, *AP2-EREBP*, and *bHLH*, while the *ClRC* gene was not associated with any transcription factors. We also analyzed the metabolic pathways associated with the target gene pool of DEGs related to rind coloration, as well as the coordinated biological functions of these differential genes across various organisms. A pathway-based analysis enhanced our understanding of the biological roles of these genes, and the obtained results of the KEGG analysis (**Supplementary Table S7**) depicted three major pathways, e.g., biosynthesis of secondary metabolites (ko01110), metabolic pathways (ko01100), and ribosome function (ko03010), that might play key roles in regulating the rind coloring process.

### RT-qPCR analysis for functional verification of key DEGs

**Figure 8:**
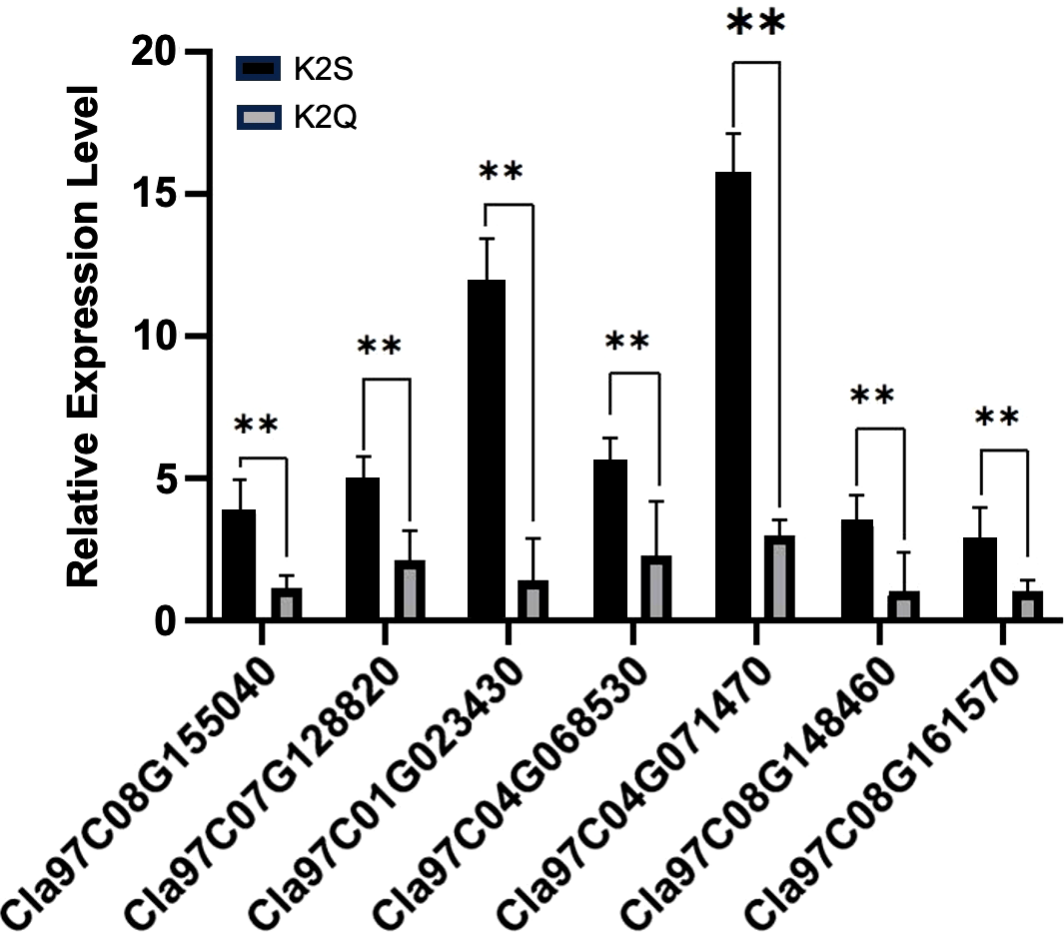
The relative expression pattern of identified DEGs and candidate gene modulating differentiated fruit rinds of contrasted parent lines at 14 days after pollination (DAP). Asterisks (**) represent an extremely significant difference.

Then, we screened pathway entries that share the same biological function as the *ClRC* gene (ko01100//metabolic pathways, ko01110//biosynthesis of secondary metabolites, ko00130//Ubiquinone and other penoid-quinone biosynthesis). This was combined with the raw RNA-seq data of genes exhibiting higher expression levels and differences in trends from 7 to 14 DAP. We ultimately selected key genes (*C01G023430*, *C04G071470*, *C09G165830*, *C07G128820*, *C08G148460*, and *C08G155040*) and checked their functional verification based on the RT-qPCR verification using fruit rinds collected at 14 DAP. As illustrated in **Figure 8**, it was noticed that the gene expression levels in the dark green peel lines were significantly higher than those in the light green peel lines, exhibiting the same expression pattern as the *ClRC* gene. This suggested that genes with similar biological functions may also play a role in the rind coloring process.

## Discussion

Watermelon is known as a major fruit crop of the Cucurbitaceae family that represents an incredible diversity of traits (Pan et al., 2020). To date, several molecular genetics and breeding studies have been conducted for the detection of candidate genes regulating qualitative and quantitative traits of watermelon (Amanullah et al., 2022, 2023), but the genetic regulation mechanism involved in the rind color trait in watermelon has not been well studied. Hence, research on gene regulatory mechanisms affecting rind color is getting good attention in recent days.

In this study, we utilized the segregated F_2_ mapping populations derived from the two different lines of watermelon (K2S line with dark green rind and K2Q line with the light green rind) (**Figure 1**). The physiological analysis showed an obvious difference in photosynthetic pigment contents (chlorophyll and carotene) of fruit rinds of both parental lines (**Table 1**). Genetic inheritance analysis exhibited the perfect segregation ratio, indicating that dark green rind color in watermelon is governed by a single dominant gene (**Table 2**). The statistical analysis revealed that the numbers of fruits with dark green and intermediate rinds significantly exceeded those of light green rinds, suggesting that dark green rind color has a substantial impact, while light green rind color exhibited incomplete dominance. This result is aligned with previous research on cucumbers and watermelons (He et al., 2023; Poole, 1944; Weetman, 1937). However, we observed that differentiation between dark and light green rind colors can be identified at 14 DAP, and the critical transition period occurred between 7 to 14 DAP (**Figure 2**), with obvious differences in the internal chloroplast structure of contrasted parental lines (**Figure 3**).

Further, the candidate genetic locus of dark green rind color was found in 37.52 kb region positioned on chromosome 8, based on utilizing the whole genome BSA-seq technology (**Figure 4**). Fine mapping of targeted *ClRC* locus exposed a candidate *Cla97C08G161570* gene, and a single nsSNP in the CDS region was identified between the K2S and K2Q mutants, occurring in the fifth exon of the gene (Chr8:27,994,761, C→G). This base mutation caused a change in the translated amino acid at position 171 in the protein sequence from glycine (G) to arginine (R) (**Figure 5**). Thus, *Cla97C08G161570* was predicted as the target gene, which encodes the 2-phytyl-1,4-*β*-naphthoquinone methyltransferase protein (**Supplementary Table S4**). A BLAST search of the Arabidopsis database found that the *At5G22350* gene had the most similarity with the *Cla97C08G161570* gene (**Figure 6**). We noticed that fine genetic mapping results and identified candidate gene(s) are consistent with the previous study of Li et al., 2019; however, the developed segregating mapping populations were derived from different parental lines. In addition, we investigated the fruits of 144 natural GWAS populations of watermelon and checked the base change at locus of Chr8:27994761 (V2_97103 genome) (**Supplementary Table S1**). A total of 40 fruit lines were identified with dark green rinds; among those, 27 lines with dark green rind depicted G base mutation (**Supplementary Figure S1**), while 2 and some other fruit lines with dark green rind exhibited C base mutation at the same site (**Supplementary Figure S2**). Of the 102 lines with light green rind, a total of 22 fruit lines exhibited G base mutation (**Supplementary Figure S3**), whereas the remaining 80 lines have a C base mutation. The base change from C to G at the candidate genomic site (Chr8:27994761) of the *Cla97C08G161570* gene was assumed to be related with the transformation of watermelon rind color from light green to dark green, a phenomenon commonly observed in natural populations. However, there were 2 lines with dark green rind that retained a C base at this site, and 22 lines with light green rind showed G base mutation, among which 13 are derived from wild watermelon materials. These finding suggested the potential existence of different regulatory genes influencing rind color in these wild-type materials, justifying further functional investigation.

Numerous studies have been performed for identifying the candidate genes regulating the rind color in Cucurbit crops. In cucumber (*Cucumis sativus* L.), there are seven types of rind color: dark green, green, light green, white-green, yellow-white, and milky white, and it is significantly influenced by environmental factors (Cui et al., 2023). The molecular studies identified a candidate gene (*Csa7G051430*) located on chromosome 7, the ortholog of Arabidopsis *ARC5*. This *ARC5* is known to limit chloroplast division, and RNA interference of this gene results in a reduction in chloroplast number and an increase in chloroplast volume (Gao et al., 2003; Qian et al., 2015). Similarly, it was discovered that the *Csa6G133820* gene, which carries a pathogenic SNP, encodes a Ycf54-like protein and is associated with the cyclase step of chlorophyll biosynthesis. The subcellular position of genes in chloroplasts further supports their role in chlorophyll biosynthesis. In summary, these studies indicate that *CsaARC5* and *CsYcf54* are contributing to modulating the light green peel characteristic of cucumber (Lun et al., 2016). The recombinant inbred lines (RIL) exhibiting green and white rinds were used for the identification of candidate genes responsible for rind color on chromosome 3, and three key genes (*Csa3G904080*, *Csa3G904100*, *Csa3G903500*, and *Csa3G902950*) were predicted (Dou et al., 2018). It was also reported that the white-green peel of cucumber is a recessive trait governed by a single gene “w,” which is linked to *Csa3G904140* on chromosome 3 (Yang et al., 2018). An insertion of a single nucleotide polymorphism (SNP) affects the stop codon, thus influencing fruit performance, and the *w* gene plays a role in regulating the white rind color of cucumber (Liu et al., 2016; Tang et al., 2018). Furthermore, *CsMYB36*, which regulates yellow-green peel (Ning et al., 2018), and *CsMYB60*, responsible for orange peel, have similarly been known (Li et al., 2013; Liu et al., 2019). Currently, cucumber peel color traits have been associated with six genes: green (*gn*), light green (*lgf*), orange (*R*), yellow-green (*yg*), light green (*lgp*), and white (*w*). Although numerous studies have reported the discovery of genes related to cucumber peel color traits, the molecular regulatory mechanisms remain inadequately understood (Miao et al., 2011).

Pumpkin (*Cucurbita moschata* L.) exhibits a wide range of types and displays various genetic mechanisms of peel color phenotypes, including *B* (bicolor), *Y* (yellow fruit color), *W* (weak fruit coloration), *l-2* (light pigmentation on fruit-2), *l-1* (light fruit color-1), and *D* (dark green stem). The bicolor gene (*B*) is an early-maturing gene associated with yellow skin, which can interact with other genes; together with *l-1* and *l-2*, it determines the degree of yellow coloration. The *Y* gene is the primary gene influencing the transformation of the peel color from yellow to green, with an interaction between *B* and *Y* that exhibits an epistatic effect (Paris et al., 2004). The cucumber and the Chinese pumpkin variety were used as research materials, and peroxidase (POD) and esterase (EST) isoenzymes and stomatal morphology were evaluated, which indicated a clear distinction between green and yellow-green phenotypes (Li et al., 2007). Further, it was proposed that green is dominant over yellow rind color and the green rind gene (*Gr*) is located on linkage group 5 (Gong et al., 2008). Moreover, *B* also interacts with *W* to influence the cream-colored peel phenotype (Roe and Emis, 1977). A molecular investigation revealed that fruit skin color is a trait governed by a nuclear gene. This quality trait exhibits complete dominance between non-all-green and all-green phenotypes and incomplete dominance between red and green skins. Finally, the seventh linkage group was identified, containing a gene responsible for all-green skin color, designated as *gf* (Xiang, 2013). It was proposed that pumpkin peel color is regulated by a single gene locus (*qpc8-a*) positioned on linkage group 8, controlling light green spotted peel, which is dominant over dark green spotless peel (Zhong et al., 2017). It was discovered that the traits of light green versus dark green peel, as well as spotted versus spotless peel, may be modulated by a dominant and single gene located within a 145.13 kb interval on chromosome 3 (Zhou et al., 2018). Generally, the *D-l-1* and *D-l-2* genes are the principal regulators of the transition from light green to dark green pumpkin peel (Globerson, 1969; Ge et al., 2015).

In melon, the outer rind color is mainly classified into 17 categories (Cui et al., 2023). The hybridization and derived segregated populations revealed that dark green rind is dominant over white peel, defining the major recessive gene ‘*w*’ modulating the white rind. In addition, genetic loci (*CmRc*) governing gray-green rind have been identified (Hughes, 1948). The research was conducted by integrating a hybrid combination of three different rind surface colors that ranged from green to off-white and white, with yellow being incompletely dominant over green, white, and off-white, while flower skin color and results exhibited dominance across different skin colors, though it is also incompletely dominant (Wu and Lin, 1992; Li, 2003). A genetic map was developed using experimental materials of biparental segregated F_2_ and double haploid lines (DHLs) populations. The segregation patterns of green and orange skin colors seemed to be complex and influenced by polygenic nature with epistatic effects (Monforte et al., 2004). Further, three main-effect loci were identified that were associated with the genetic control of rind color of melon (Cuevas et al., 2010). Later, it was discovered that the yellow rind of melon is regulated by the F-box protein gene (*MELO3C011980*), named *CmKFB*, which regulates the formation of naringin chalcone, thereby controlling the development of yellow rind (Feder et al., 2015; Gur et al., 2017). The study also proposed that melon rind color is regulated by two pairs of genes, with green exhibiting a dominant epistatic effect over white and white being dominant over yellow. These genes were identified at 5.68 Mb on chromosome 4 and 9.42 Mb on chromosome 10 (Ou et al., 2019). Although studies identified the candidate genetic loci and underlying genes related to melon rind color, the research on the various regulatory mechanisms remains relatively slow.

In this study, we also did comparative transcriptomic sequencing of samples collected at 0, 7, and 14 DAP, which determined that the *ClRC* gene was not annotated within the transcription factor family. So, the candidate genes associated with the rind coloring process were identified based on DEGs analysis and trend evaluation during the critical color transition period (**Supplementary Table S6**, **Figures 6**, **7**). In addition, GO enrichment analysis revealed significant biological processes related to the development of the dark green rind phenotype, primarily involving plastids, thylakoids, and the generation of precursor metabolites and energy (**Figure 7C**). KEGG pathway enrichment analysis highlighted metabolic pathways pertinent to the formation of the dark green peel phenotype, including general metabolic pathways, the biosynthesis of secondary metabolites, ribosome function, and photosynthesis (**Supplementary Table S7**). The development and regulation of fruit rind color is a multifaceted biological process involving several metabolic pathways (Matsumoto et al., 2004). Chlorophyll is the primary pigment responsible for the coloration of watermelon skin, with higher chlorophyll content observed in dark green skin compared to light green skin (Yang et al., 2024). Herein, through homology analysis and comparison (**Figure 6B**), we determined that *Cla97C08G161570* is an ortholog of the *MENG*-like gene found in Arabidopsis, that influences the color of watermelon rind. The *MENG* gene plays an essential role in the biosynthesis of phylloquinone (vitamin K1) in Arabidopsis that encodes a 2-phytyl 1,4-*β*-naphthoquinone methyltransferase. This enzyme catalyzes the final step of phylloquinone biosynthesis, whereby 2-phytyl 1,4-*β*-naphthoquinone converts into vitamin K1. Further, transcriptome analysis revealed the significant pathways mainly involved in the biosynthesis of photosynthetic pigments and enzymatic reactions (**Figure 7**). However, the content of photosynthetic pigments is influenced by metabolic pathways, genes related to plastid development, and abiotic stresses, such as light, temperature, and drought.

## Conclusion

Our research findings exposed a stable genomic locus (*ClRC*) and candidate gene (*Cla97C08G161570*) located on chromosome 08, controlling dark green rind color of watermelon based on fine genetic mapping and transcriptomic analysis. In addition, key DEGs (*C01G023430*, *C04G071470*, *C09G165830*, *C07G128820*, *C08G148460*, and *C08G155040*) exhibited significant pathways (metabolic pathways, biosynthesis of secondary metabolites, ribosomes, and photosynthesis) along with expression levels similar to that of the *Cla97C08G161570* gene, suggesting their potential involvement in modulation of dark green rind color of watermelon. We believe that the obtained results would provide additional insights in horticultural genetics and breeding studies aimed at the production of improved watermelon cultivars.

## Data Availability

The data presented in the study are deposited in the NCBI repository, accession number PRJNA1206455.
