# Supplementary material for: Uncovering the genetic mechanism of rind color trait in watermelon using fine mapping and comparative transcriptomic analysis
Source: Front Plant Sci. 2025 Mar 13;16:1553166. doi: 10.3389/fpls.2025.1553166 (PMC11965938; doi:10.3389/fpls.2025.1553166)
Supplement: Supplementary file 1 [file DataSheet1.zip › Supplementary Material/Supplementary Figures (S1-S3).docx]

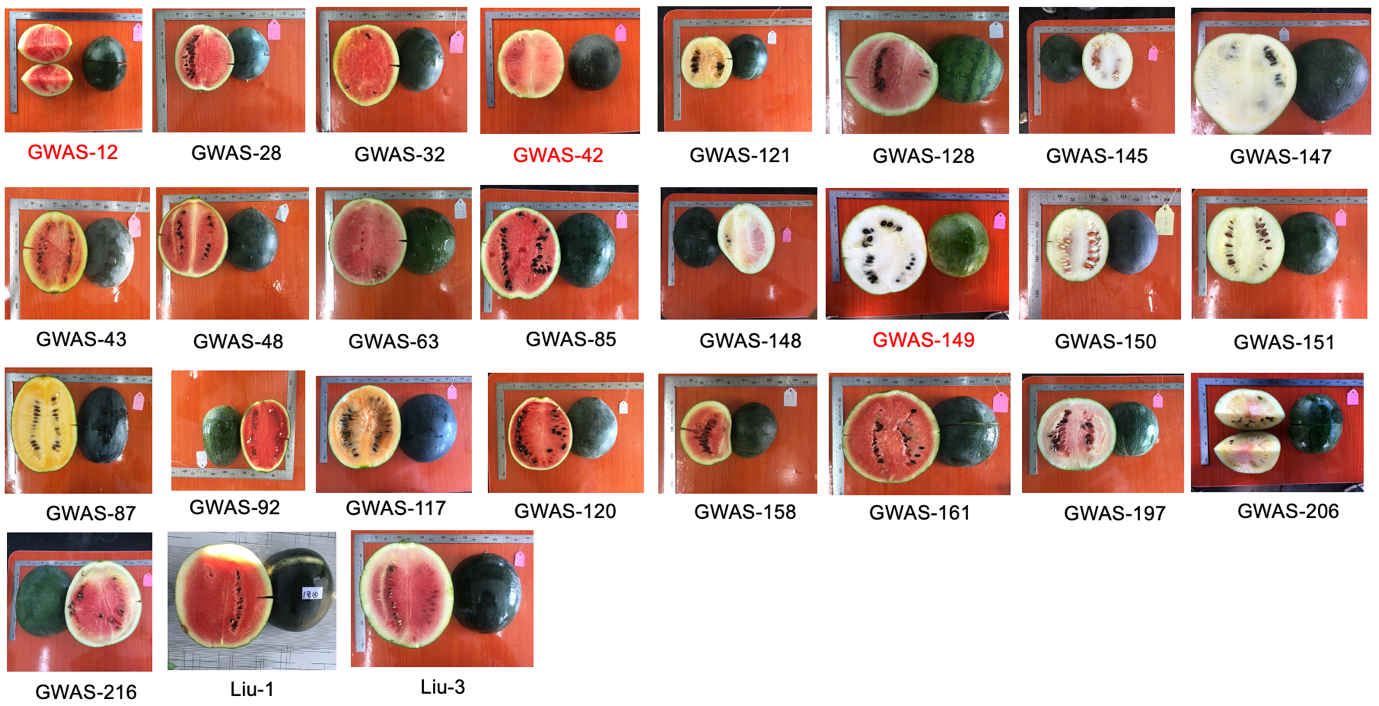


**Supplementary Figure S1 |** A total of 27 watermelon lines with dark green rind from natural population species resources depicted G base mutation at genomic position of 27994761.


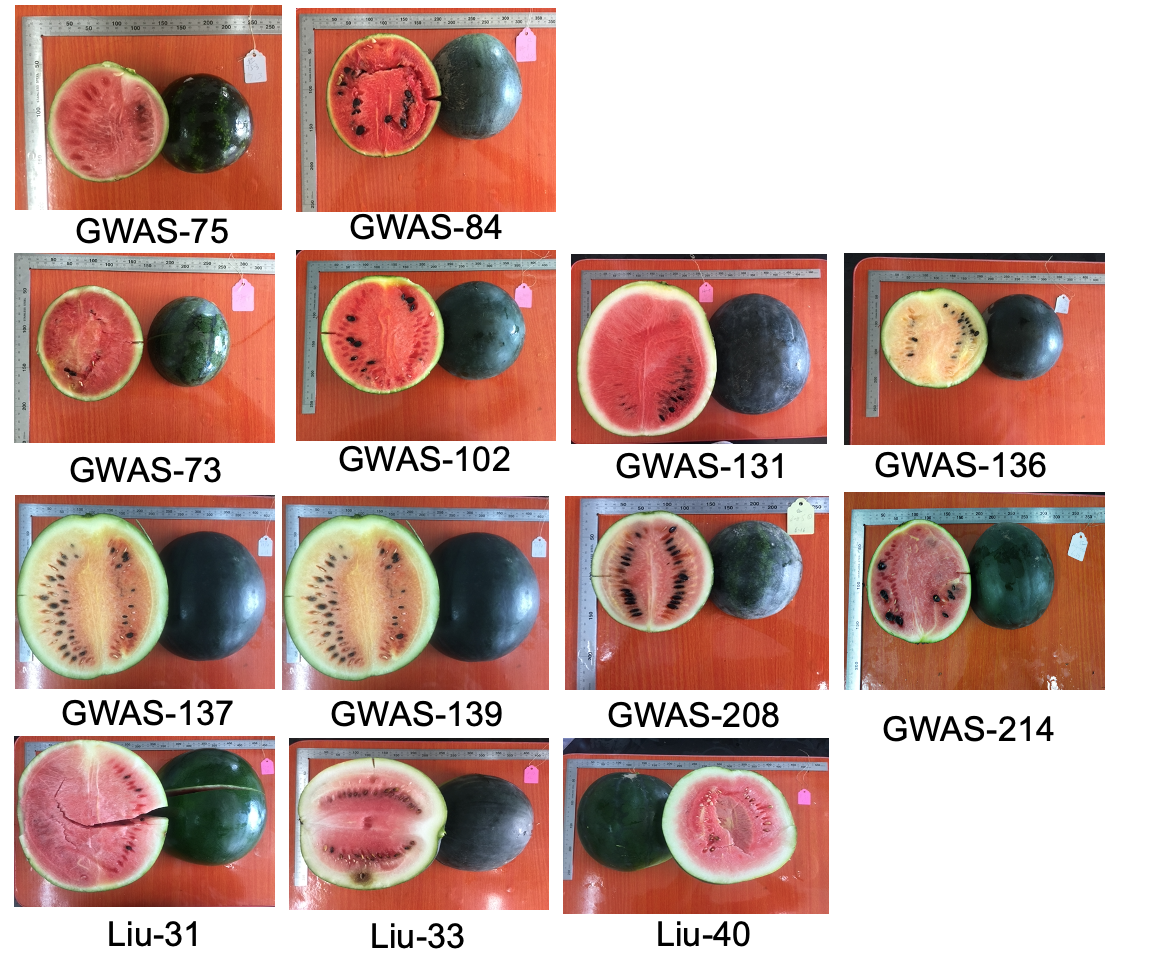


**Supplementary Figure S2 |** A total of 2 and some other watermelon lines with dark green rind from natural population species resources depicted C base mutation at genomic position of 27994761.


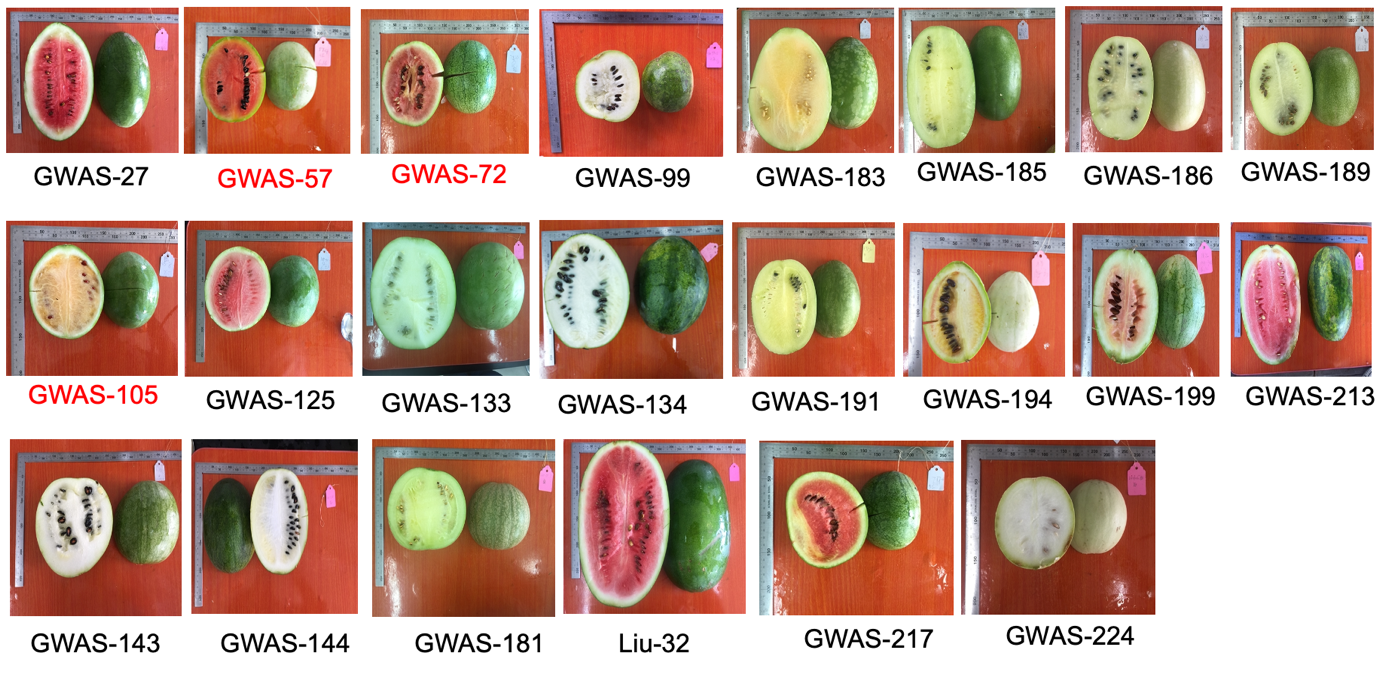


**Supplementary Figure S3 |** A total of 22 watermelon lines with light green rind from natural population species resources depicted G base mutation at genomic position of 27994761.
